# Prostaglandins regulate humoral immune responses in *Aedes aegypti*

**DOI:** 10.1371/journal.pntd.0008706

**Published:** 2020-10-23

**Authors:** Ana Beatriz Ferreira Barletta, Thiago Luiz Alves e Silva, Octavio A. C. Talyuli, Tatiana Luna-Gomes, Shuzhen Sim, Yesseinia Angleró-Rodríguez, George Dimopoulos, Christianne Bandeira-Melo, Marcos H. Ferreira Sorgine

**Affiliations:** 1 Laboratório de Bioquímica de Artrópodes Hematófagos, Instituto de Bioquímica Médica Leopoldo De Meis, Programa de Biologia Molecular e Biotecnologia, Universidade Federal do Rio de Janeiro, Rio de Janeiro, Brasil; 2 Instituto Nacional de Ciência e Tecnologia em Entomologia Molecular (INCT-EM), Brasil; 3 Departamento de Ciências da Natureza, Instituto de Aplicação Fernando Rodrigues da Silveira (CAp-UERJ), Universidade do Estado do Rio de Janeiro, Rio de Janeiro, Brasil; 4 W. Harry Feinstone Department of Molecular Microbiology and Immunology, Bloomberg School of Public Health, Johns Hopkins University, Baltimore, Maryland, United States of America; 5 Laboratório de Inflamação, Instituto de Biofísica Carlos Chagas Filho, Universidade Federal do Rio de Janeiro, Rio de Janeiro, Brasil; University of Queensland, AUSTRALIA

## Abstract

Prostaglandins (PGs) are immuno-active lipids that mediate the immune response in invertebrates and vertebrates. In insects, PGs play a role on different physiological processes such as reproduction, ion transport and regulation of cellular immunity. However, it is unclear whether PGs play a role in invertebrate's humoral immunity, and, if so, which immune signaling pathways would be modulated by PGs. Here, we show that *Aedes aegypti* gut microbiota and Gram-negative bacteria challenge induces prostaglandin production sensitive to an irreversible inhibitor of the vertebrate cyclooxygenase, acetylsalicylic acid (ASA). ASA treatment reduced PG synthesis and is associated with decreased expression of components of the Toll and IMD immune pathways, thereby rendering mosquitoes more susceptible to both bacterial and viral infections. We also shown that a cytosolic phospholipase (PLAc), one of the upstream regulators of PG synthesis, is induced by the microbiota in the midgut after blood feeding. The knockdown of the PLAc decreased prostaglandin production and enhanced the replication of Dengue in the midgut. We conclude that in *Ae*. *aegypti*, PGs control the amplitude of the immune response to guarantee an efficient pathogen clearance.

## Author summary

Mosquito immune responses work on an on/off switch model, where the recognition of microorganisms turns the switch on, and its clearance turns it off. Besides pathogen derived molecules, no other compounds are known to directly regulate the activation and the amplitude of immune responses. Here we described that prostaglandins, lipid mediators of the immunity in vertebrates, also modulate the amplitude of immune responses in mosquitoes as well. Prostaglandins regulate the production of antimicrobial peptides and other effector molecules and directly impact the susceptibility of mosquitoes to bacterial and viral infections. When prostaglandin production is impaired, immune activation is inefficient and renders the mosquito more susceptible to bacterial and Dengue infections.

## Introduction

Insects fight infections using humoral and cellular responses. Humoral immune responses comprise the secretion of compounds into the hemocoel. Among those hemolymph-soluble components there are antimicrobial peptides (AMPs), proteolytic enzymes that mediate melanization and coagulation, and control signal transduction, like the activation of the Toll pathway [1]. In cellular immune responses, hemocytes are the main players and mediate phagocytosis, nodulation and encapsulation of microorganisms [2]. Humoral and cellular immune responses are interconnected, since hemocytes produce and secrete humoral components, such as AMPs, CLIP-domain serine proteases and pro-phenoloxydases. Invertebrate immune responses work on an on/off switch model, where the recognition of pathogen-associated molecular patterns (PAMPs) turns the switch on, and its clearance turns it off. In mosquitoes, besides PAMPs, no other molecules are known to directly regulate the activation and the amplitude of immune responses. Toll, the broadly conserved NF-κB pathway, *Immune Deficient* (IMD) and the *Janus Kinase/Signal Transducer and Activator of Transcription* (Jak-STAT) are the main immune pathways activated in the mosquito in response to bacteria, fungal and viral infections [3]. Activation of Toll and IMD leads to the production of AMPs and other effector molecules that participate in the pathogen killing [2]. In mosquitoes, the activation of Jak-STAT increases nitric oxide synthase (NOS) expression and culminates with *Plasmodium* killing [4].

Prostaglandins (PGs) are bioactive lipids derived from arachidonic acid and in insects were originally implicated in egg-laying and reproduction [5, 6]. Later, PGs were recognized as mediators of immune responses in the tobacco hornworm *Manduca sexta* [7, 8], the kissing bug *Rhodnius prolixus* [9], the beet armyworm *Spodoptera exigua* [10] and the mosquito *Anopheles gambiae* [11]. PGs trigger hemocyte spreading behavior, nodule formation, AMP expression and melanization cascade, participating in cellular and humoral responses. Besides reproductive organs, Malpighian tubules and salivary glands [5, 12, 13], prostaglandins can be produced by the midgut tissue in response to the microbiota [11, 14]. Midgut prostaglandins attract hemocytes and are the first signal necessary to establish immune memory in *An*. *gambiae* [11]. More recently, the first insect PGE2 receptor (MansePGE2R) was characterized in hemocytes of *M*. *sexta*, opening a new venue for eicosanoid signaling pathway studies in insects [15].

Enzymes from the phospholipase A2 family mediate the first and limiting step for the biosynthesis of PGs [16]. This enzyme cleaves phospholipids containing C20 polyunsaturated fatty acids (PUFAs) releasing arachidonic acid, the main precursor of several bioactive lipids, including PGs. The activity of these enzymes is sensitive to immunomodulators like PAMPs and cytokines [17]. Puzzlingly, cyclooxygenases, the enzymes that use arachidonic acid to generate PGs, are absent in the genome of insects [18]. In *An*. *gambiae* and the fruit fly *Drosophila melanogaster*, heme peroxidases functionally replace the cyclooxygenases activity necessary for PG synthesis [11, 19]. Classical inhibitors of PG synthesis in mammals, such as acetylsalicylic acid (ASA) and ibuprofen, can impair the production of PG in insects, being able to inhibit heme peroxidases as well [19].

Although PGs have previously been implicated in the regulation of insect immune responses, their global effect on gene expression of immune components and its implication in viral susceptibility have not been addressed. Here, we show for the first time that, blocking of PG production compromises humoral immune responses in the mosquito *Ae*. *aegypti*, downregulating Toll and IMD pathways. As a result, mosquitoes become more susceptible to bacteria and Dengue virus infections. We also characterized one more component of PG synthesis in mosquitoes, describing that a cytosolic phospholipase A2 (PLA2c) induced by the microbiota is involved in PG synthesis in the midgut. Knockdown of this PLA2c increases mosquito susceptibility to bacteria and Dengue infections.

## Methods

### Mosquitoes and cell culture

*Aedes aegypti* (Red Eye strain) were raised in an insectary at the Federal University of Rio de Janeiro, Brazil, under a 12 h light/dark cycle at 28°C and 70–80% relative humidity. Larvae were fed with dog chow. Adults were maintained in cages and fed a solution of 10% sucrose *ad libitum*. Four- to seven-day-old females were used in the experiments. *Aedes aegypti* Aag-2 cells were maintained at 28°C in Schneider´s *Drosophila* medium with L-glutamine (Life Tecnologies, Grand Island, NY) supplemented with 10% Fetal Bovine Serum (FBS) (Cultilab, Campinas, SP) and penicillin (100 u/mL) and streptomycin (100 μg/mL) (LGC biotecnologia, Cotia, SP).

### *In vitro* bacterial and viral infection

For bacterial challenge, cells were incubated with two different heat-killed bacteria as previously described [20]: *Micrococcus luteus*, a Gram-positive bacteria, and *Enterobacter cloacae*, a Gram negative bacteria. Aag-2 cells were incubated with 100 bacteria per cell (10^7^ bacteria/ well), either heat killed or live, as described for each experiment. Cells were also incubated with 0.5 mg/mL Zymosan A (Sigma-Aldrich, St. Louis, MO), as previously described [21]. For viral infection, cells were infected with Sindbis virus or Dengue virus (DENV) using a MOI (Multiplicity of Infection) of 1, as previously described [22].

### Mosquito meals

Mosquitoes were artificially fed with different diets: (1) 10% sucrose (*ad libitum*), (2) rabbit blood, (3) bicarbonate-buffered saline-agarose (BBSA) supplemented with *Serratia marcescens* (Sm). The BBSA solution was composed of glucose (10 mg), 500 mM freshly made bicarbonate buffer pH 7.4 (10 μL), 0.5 mg low melting-point agarose and 100 mM ATP, pH 7.4 (5 μL). The final volume was set to 500 μL with 150 mM NaCl. Feeding was performed using water-jacketed artificial feeders maintained at 37°C and sealed with Parafilm “M” (Sigma-Aldrich, St. Louis, MO) membrane. For depletion of mosquito’s microflora, females were fed with sucrose 10% supplemented with antibiotics, penicillin (100 u/mL) and streptomycin (100 μg/mL) (LGC biotecnologia, Cotia, SP) for 4 days, as previously described [23].

### Mosquito Dengue infections and titration by plaque assay

The New Guinea C (NGC) DENV-2 strain was propagated in *Aedes albopictus* C6/36 cells: cells seeded to 80% confluency in 75 cm^2^ flasks were infected with virus stock at a multiplicity of infection (MOI) of 3.5, and incubated for 6 days at 32°C and 5% CO_2_. Infected cells were scraped into solution and lysed to release virus particles by repeated freezing and thawing in dry ice and a 37°C water bath and centrifuged at 12.000g for removal of cell debris. Virus suspension was mixed 1∶1 with commercial human blood and supplemented with 10% human serum. The bloodmeal was maintained at 37°C for 30 min and then offered to mosquitoes via an artificial membrane feeding system, as described above. After seven days post infection, individual midguts were homogenized in DMEM with a Bullet Blender (NextAdvance), serially diluted, and then inoculated onto BHK cells seeded to 80% confluency in 24-well plates (100 ul per well) for viral titration. Plates were rocked for 15 min at room temperature, and then incubated for 45 min at 37°C and 5% CO_2_. Subsequently, 1 mL of DMEM containing 2% FBS and 0.8% methylcellulose was added to each well, and plates were incubated for 5 days at 37°C and 5% CO_2_. Plates were fixed with a methanol/acetone mixture (1∶1 volume) for >1 h at 4°C, and plaque-forming units were visualized by staining with 1% crystal violet solution for 10 min at room temperature.

### Microarray gene expression analysis

Aag-2 cells were seeded to a confluence of 80% in 12-well plates and treated in quadruplicate with the following: (a) Heat-killed Gram negative bacteria *Enterobacter cloacae*; (b) Heat-killed Gram negative bacteria *Enterobacter cloacae* in the presence of 1.5 mM of Acetylsalicylic acid (ASA); or (c) Schneider´s *Drosophila* medium.

After incubation at 28°C, for 6 hours of conditions (a) and (b), infected and control cells were lysed by the addition of 600 μL of Buffer RLT (Qiagen) and homogenized for 30 s with a rotor-stator homogenizer. RNA was then extracted with the Qiagen RNeasy Mini Kit. Two micrograms of total RNA were used for synthesis of Cy3- and Cy5-labeled cRNA probes and hybridizations were carried out on an Agilent-based microarray platform. Hybridization intensities were determined with an Axon GenePix 4200AL scanner, and images were analyzed with Gene Pix software. Expression data were processed and analyzed as previously described [24, 25]. Self–self-hybridization has been used to determine the cutoff value for the significance of gene regulation on these types of microarrays to 0.78 in log2 scale, which corresponds to 1.71-fold regulation [26]. Numeric microarray gene expression data are presented in S1 Dataset.

### RNA Extraction, cDNA synthesis and Quantitative PCR

Total RNA from cells and mosquitoes in all conditions was extracted using the TRIZOL reagent (Invitrogen, St. Louis, MO) following the manufacturer's instructions. RNA was treated with DNAse I (Fermentas, Waltham, MA) and first-strand cDNA synthesis was carried out using High-Capacity cDNA Reverse transcription kit (Applied Biosystems, St. Louis, MO). The amplification efficiency of the experimental set for each gene was tested with serial dilutions of cDNA and was only used if the resultant efficiency was ≥ 90%. Each PCR reaction (15 μL volume) contained diluted cDNA, Power SYBR Green PCR Master Mix (Applied Biosystems, St. Louis, MO) and 300 nM of forward and reverse primers. Quantitative PCR was performed in a StepOnePlus Real Time PCR System (Applied Biosystems, St. Louis, MO) using Applied Biosystems recommended qPCR conditions (20 seconds at 95°C followed by 40 cycles of 95°C for 1 second and 20 seconds at 60°C followed by a melting curve to assure a single product was amplified). The comparative ΔΔCt method was used to evaluate changes in gene expression levels and all standard errors were calculated based on ΔCt as described in Applied Biosystems User Bulletin #2 (http://www3.appliedbiosystems.com/cms/groups/mcb_support/documents/generaldocuments/cms_040980.pdf). The *A*. *aegypti* ribosomal protein 49 gene, RP-49, was used as endogenous control (accession number AAT45939), based on previous data [27]. Each figure represents at least five biological replicates with three technical replicates for each sample. Primers used in this manuscript for gene knockdown and expression were listed in S1 Table. For 16S expression in mosquito midguts the following primers were used: 16S forward: TCCTACGGGAGGCAGCAGT and 16S reverse: GGACTACCAGGGTATCTAATCCTGTT.

### Quantification of prostaglandins in tissues and cell supernatant

For quantification of prostaglandins, Aag2 cells were incubated in Hanks Balanced Salt Solution with calcium and magnesium (HBSS ++). Cells were also incubated with 1.5 mM ASA in HBSS++ for 1 hour and then the heat-killed bacteria *Enterobacter cloacae* was added to the media. After one or 24 hours, the supernatant was collected and centrifuged at 12.000 rpm to remove cells in the supernatant before the measurement. For tissue quantification of prostaglandins, 10 midguts were dissected and placed in HBSS++ for 1 hour at 28°C. After this, tissues and the medium were collected, homogenized with a pestle and the homogenate was centrifuged at 12.000 rpm. The supernatant was used for measurement. Prostaglandins were quantitated by a commercial EIA kit (Prostaglandin Screening kit, Cayman Chemical Co., Ann Arbor, MI) according to the manufacturer´s instructions. The prostaglandin screening ELISA kit has a range of detection of 15.6–2000 pg/ml. All the measurements conducted were done with a standard curve to allow the calculations of prostaglandin levels by interpolating the values within the curve. So, the levels of prostaglandins detected are inside the range of detection which relates to the sensitivity of the assay. Alternatively, PG synthesis within Aag2 cells was immunolocalized by EicosaCell assay [28]. Briefly, cells were mixed with water-soluble 1-ethyl-3-(3-dimethylamino-propyl) carbodiimide (EDAC; 0.2% in HBSS containing 1% BSA for 10 min) (Sigma), used to cross-link eicosanoid carboxyl groups to amines in adjacent proteins, and then washed, cytospun onto glass slides and subjected to a blocking step (1%BSA for 30 min), were incubated with rabbit anti-PGs Abs (Cayman Chemicals) overnight and secondary DyLight546 red fluorochrome anti-rabbit IgG (Jackson ImmunoResearch Laboratories) for 1 h. Mounting medium containing DAPI was applied to each slide before coverslip attachment to allow visualization of blue-stained eosinophil nuclei. Images were obtained using an Olympus BX51 fluorescence microscope at 100x magnification and photographs were taken with the Olympus 72 digital camera (Olympus Optical Co., Tokyo, Japan) in conjunction with CellF Imaging Software for Life Science Microscopy (Olympus Life Science Europa GMBH, Hamburg, Germany).

### Bacteria proliferation assay and quantitation of Dengue and Sindbis virus

Aag2 cells were placed in a plate and preincubated with ASA 1.5 mM for 2 hours. After this, live *Enterobacter cloacae* were added to the media and after 4 hours of co-incubation, an aliquot of the supernatant was plated in LB agar medium. Plates were placed in an incubator overnight at 37°C. The number of colonies was counted to evaluate the growth of the bacteria in the supernatant of treated and non-treated cells. Alternatively, Aag2 cells were infected with Dengue or Sindbis virus (Halsted strain or New Guinea C strain) and, after 4 days of infection, an aliquot of the supernatant was collected and RNA was extracted from particles present in the supernatant. Viral RNA was used to synthesize cDNA and the amount of viral RNA was measured by quantitative PCR, using SYBR Green (Applied Biosystems, St.Louis, MO). Viral RNA amount was normalized to the number of cells in each well, counted using Trypan Blue.

### Survival curves

Females were previously fed for three days with ASA 5 mM in a sugar solution, *ad libitum*. After that, females were artificially fed with a BBSA solution supplemented with *Serratia marcescens* (Sm). After growing overnight in liquid LB medium, the bacteria was pelleted, washed, re-suspended in PBS and mixed with BBSA components to a final volume of 1 mL. Fully engorged mosquitoes, taken immediately after feeding, with or without bacteria, were transferred to new cages and scored for survival at different time points.

### dsRNA synthesis and gene knockdown

Three to four day old female *Ae*. *aegypti* were cold anesthetized and injected with 69nl of a 3μg/μl dsRNA solution for each phospholipase (PLAc and PLAs). DsRNA was generated from *Ae*. *aegypti* whole body cDNA template using the MEGAscript RNAi kit (ThermoFisher Scientific, Waltham, MA, USA). Specific primers containing a T7 tail were designed for each phospholipase, PLAc (AAEL001523) and PLAs (AAEL009876), and are listed in S1 Table. A 611-bp fragment was amplified for PLAc and a 648-bp fragment was amplified for PLAs. A 218-bp fragment was amplified from LacZ gene cloned into pCRII-TOPO vector using M13 primers to generate a dsRNA control.

### Statistical analysis

All analyses were performed with GraphPad Prism statistical software package (Prism 5.01, GraphPad Software, Inc., San Diego, CA). Asterisks indicate significant differences (*p< 0.05; **p<0.01; ***p<0.001) and the type of test used in each analysis is described in its respective figure legend.

## Results

### Gram-negative bacteria and the microbiota modulate PG production in Aag2 cell line and *Aedes aegypti* midgut, respectively

**Fig 1:**
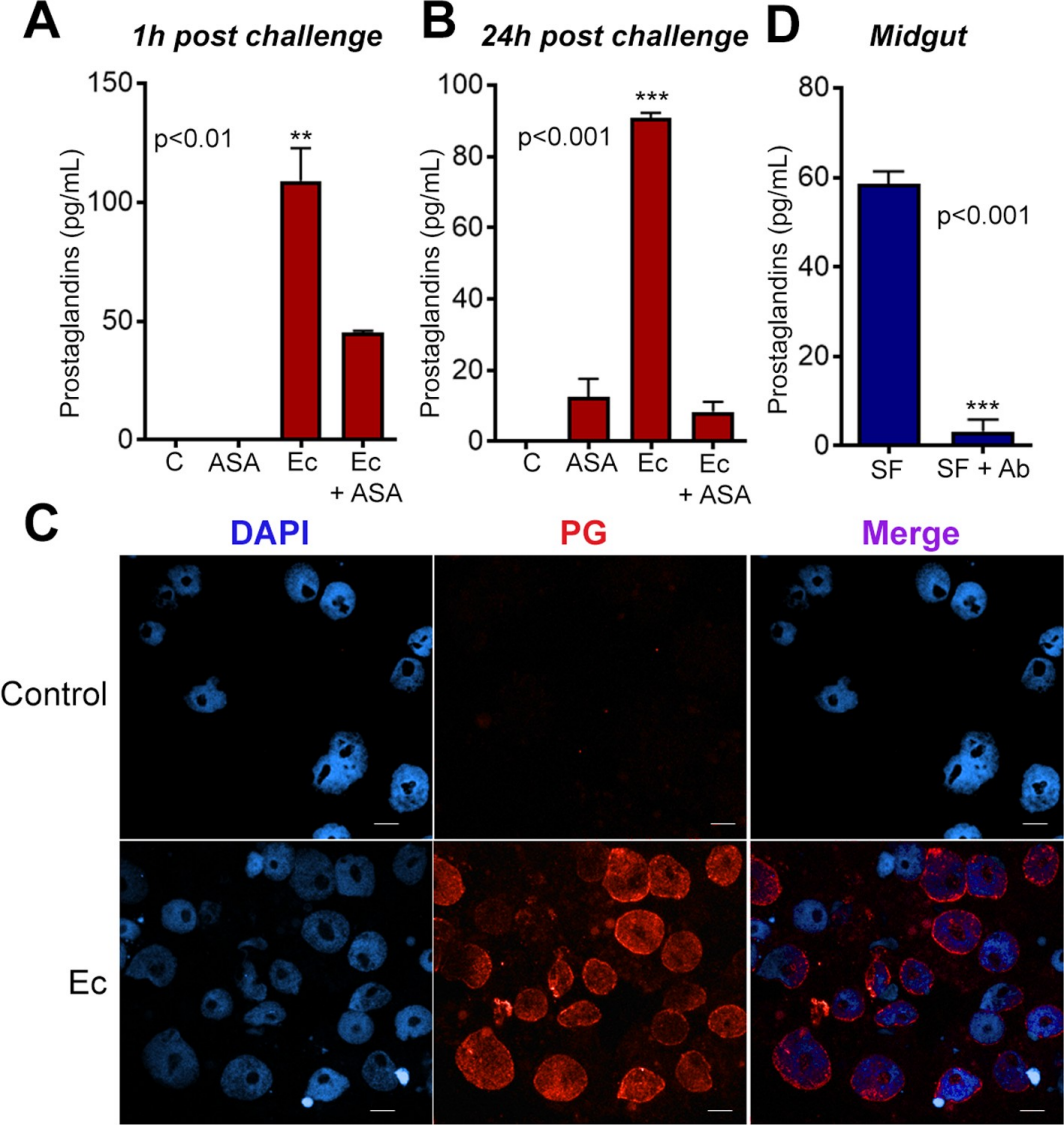
*E*.*cloacae* (Ec) stimulates PG production in Aag2 cells and *Aedes* midgut cells. (A-B) Aag2 cells were incubated with heat-killed bacteria *E*. *cloacae* (Gram negative, Ec) in the presence or absence of ASA for 1 hour (A) or 24 hours (B). PG levels in the supernatant of Aag2 cells incubated with heat killed Ec and ASA. (C) PG detection *in situ* (by EicosaCell assay) in Aag2 cells challenged with heat-killed bacteria. (D) PG levels in midguts of *Ae*.*aegypti* females sugar fed (SF) and antibiotic treated (SF + Ab). Statistical analyses were performed using ANOVA followed by Dunnett’s multiple comparison test for figure A and B. In C, statistical analyses were conducted as an unpaired t-test. Error bars represent mean ± SEM. **P≤0.01, ***P≤0.001. Scale Bars = 7μm.

We investigated whether exposing the mosquito *Ae*. *aegypti* to a bacterial challenge would increase the production and secretion of prostaglandins. To test this hypothesis, we stimulated the immune responsive *Ae*. *aegypti* cell line, Aag2, with heat-inactivated Gram-negative bacteria *E*. *cloacae* (Ec) during one or 24 h, and then measured the prostaglandin content in the culture supernatant. Prostaglandin levels were undetectable in the supernatant of non-stimulated Aag2 cells, but upon stimulation, the levels of prostaglandin increased up to 120 pg/mL after 1 h and remained elevated after 24 h (Fig 1A and 1B). In *Drosophila*, an acetylsalicylic acid (ASA)-sensitive COX-like peroxidase mediates prostaglandin synthesis [19]. We observed that Ec-induced prostaglandin secretion was sensitive to ASA in Aag2 cell line as well (Fig 1A and 1B).

To identify the subcellular sites involved with prostaglandin production, we used a previously established technique called EicosaCell [28], which consists of immunostaining of newly synthetized eicosanoid lipid mediators within cells. Aag2 cells were stimulated with heat-killed *E*. *cloacae* and, after 24 h, were stained for prostaglandin detection. In non-stimulated cells, prostaglandins were undetectable, however upon bacterial challenge, we detected prostaglandin in nuclear and perinuclear sites (Fig 1C).

Since Aag2 cells produced PGs in response to bacterial challenge, we investigated whether the *Ae*. *aegypti* midgut could produce PGs. The mosquito midgut is constantly exposed to the natural microbiota, which is composed mainly by Gram-negative bacteria [29–31], that increases up to three orders of magnitude upon blood feeding [23]. Prostaglandin levels were compared between female midguts treated with antibiotics in a regular sugar diet and untreated females. The reduction of microbiota levels strongly suppressed prostaglandin production by the midguts (Fig 1D and S1 Fig). These results suggest that Gram-negative bacteria might be important modulators of prostaglandin production in *Ae*. *aegypti*.

### Inhibition of prostaglandin production suppresses the *Ae*. *aegypti* immune response against Gram-negative bacteria and virus infections

**Fig 2:**
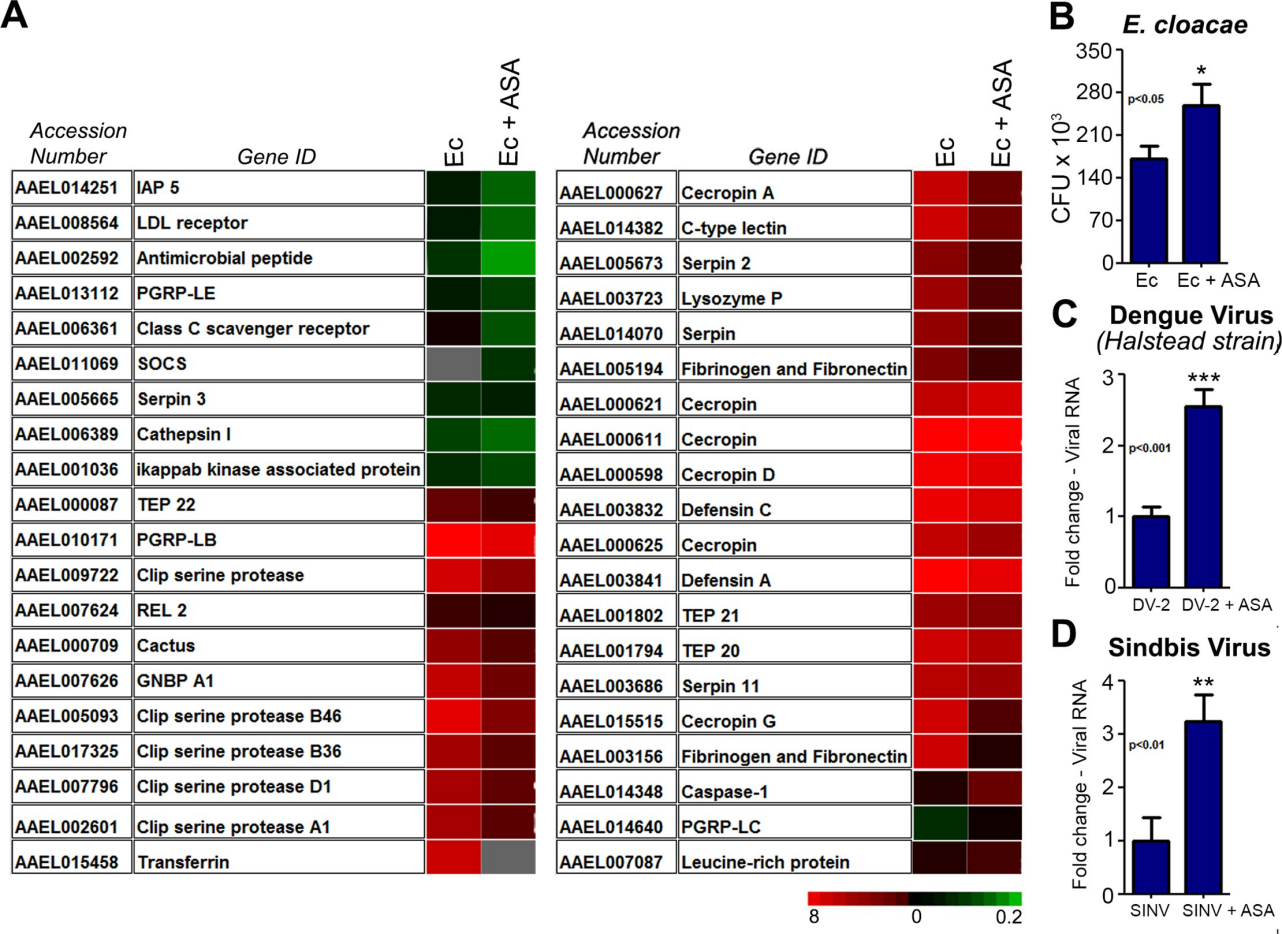
ASA treatment compromises the proper expression of immune genes and the ability to control bacterial and viral infections. (A) Immune related genes modulated by ASA treatment in the microarray analysis. Green color indicates down-regulated genes and red color is referred to up-regulated ones. Complete detailed expression data can be seen in S1 Dataset. A complete list of the immune genes regulated by ASA treatment can be seen in S2 Dataset. (B) Number of CFU recovered from the supernatant of Aag2 cells that were pre-treated or not with ASA before Ec exposure. (C-D) Viral RNA recovered from the supernatant of Aag2 cells infected with Dengue and Sindbis virus after pretreatment with Aag2 cells (C) Dengue virus RNA recovered after 4 days post infection (D) Sindbis virus RNA recovered 2 days post infection. Error bars represent mean ± SEM. Unpaired t-test, *P≤0.05, **P≤0.01, ***P≤0.001. Each biological replicate corresponds to a well on a plate and at least three independent experiments were performed per assay. Viral RNA amounts were normalized by the number of cells present in the well, which were determined using trypan blue stain.

We evaluated the impact of PG synthesis inhibition on the global gene expression in the cell line Aag2. Aag2 cells were pre-treated with ASA or vehicle for one hour, and then challenged with the heat-killed Gram-negative bacteria, *E*. *cloacae* (Ec). In vehicle-pretreated Aag2 cells, Ec-challenge induced the upregulation of 361 genes, whereas 822 were down-regulated (Fig 2A, S2 Fig and S1 Dataset). In contrast, in ASA-pretreated, Ec-challenged Aag2, 1008 genes were up-regulated and 1267 genes were down-regulated. 114 genes were commonly up-regulated and 399 down-regulated in both treatments (S2 Fig and S1 Dataset).

Next, we investigated how these treatments affected immune-related genes. In ASA-pretreated Aag2 cells, 99 immune-related genes were suppressed (S2 Dataset). Transcripts for clip-domain serine proteases were strongly down-regulated, followed by transcripts related to the toll pathway, and transcripts for protease inhibitors serpins. Some transcripts of the IMD pathway were strongly down-regulated, such as the transcriptional factor REL 2, the receptors PGRP-LE and PGRP-LB (*Peptidoglycan Recognition Protein*), and some AMPs, e.g. defensins and cecropins. The negative regulator of the Jak/STAT pathway, SOCS (*Suppressor Of Cytokine Signaling*), was up-regulated by ASA-treatment suggesting a repression of the Jak/STAT pathway. The thioester–containing proteins TEP 20, 22 and 19, putatively involved in pathogen opsonization, were also down-regulated by ASA treatment (Fig 2A). We validated the microarray results by qPCR by measuring the transcript levels of the AMPs defensin A, attacin, cecropin G and D and gambicin (S3 Fig). Following Ec challenge, the expression of all AMPs was significantly increased compared to unchallenged Aag2 cells (S3 Fig). Accordingly, to the microarray and qPCR validation, ASA pretreatment hampered the expression of defensin A, attacin and cecropin G, while the transcript levels of gambicin were unaltered (Fig 2A, S3 Fig and S1 Dataset). We also observed a reduction on the transcript abundance of defensin A and gambicin when Aag2 cells were pre-treated with ibuprofen–a competitive inhibitor of cyclooxygenase, that has a different mechanism of action from ASA (S4 Fig).

We evaluated whether ASA-treated Aag2 cells became immune deficient and, thereby, more susceptible to infections. To test this hypothesis Aag2 cells were pre-treated with either the inhibitors (ASA or ibuprofen) or vehicle (control), followed by incubation with live Ec. After 4 hours of incubation, supernatants of Aag2 cells previously treated with inhibitors presented significantly higher levels of *E*. *cloacae* in comparison to supernatant of control cells (Fig 2B and S5A Fig), suggesting a compromised ability of drug-treated cells in fighting infections. To test whether the prostaglandin inhibition-related immune suppression could also affect anti-viral defense, Aag2 cells were treated with ASA or ibuprofen prior to infection with Dengue or Sindbis viruses. In both cases, treatment with ASA increased the amount of viral RNA in the supernatant. Viral RNA from Dengue virus increased 2.5-fold while Sindbis RNA increased about 3-fold (Fig 2C and 2D) four days post infection, showing that prostaglandin production is necessary for the generation of both an antiviral and antibacterial immune response. Also, treatment with ibuprofen and infection with another strain of Dengue virus (New Guinea C) showed the same pattern, culminating with an increase of 3–4 fold in viral RNA (S5B and S5C Fig). To confirm that ASA treatment impairs PG production during a viral infection, we measured PG levels in the supernatant of Aag2 cells infected with Dengue. The levels of PG went from 17 pg/mL in the supernatant of infected cells to non-detectable levels after ASA treatment.

### Inhibition of prostaglandin production impairs antimicrobial gene expression in *Ae*.*aegypti* midgut and increases Dengue viral loads

**Fig 3:**
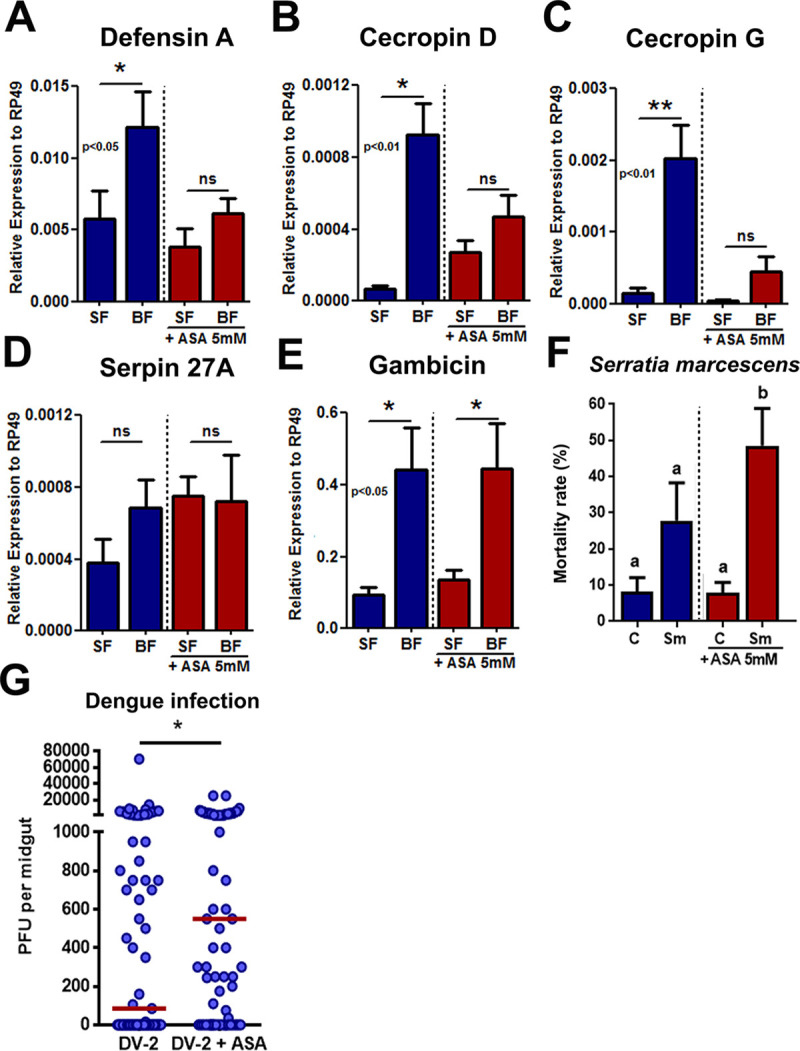
In the midgut, PG is required for proper induction of immune genes after blood feeding, and its inhibition compromises survival after bacterial infection. (A-E) *Aedes aegypti* female mosquitoes were pretreated with a sugar solution supplemented with ASA for three days, before blood feeding. Twenty -four hours after feeding midgut expression of AMPs (A) defensin A, (B) cecropin D, (C) cecropin G, (D) serpin 27A and (E) gambicin was analyzed by qPCR. Statistical analyses were performed using unpaired t-test comparing sucrose and blood groups in each condition. (F) Mortality rate of mosquitoes after feeding with the bacteria *Serratia marcescens* (5x10^8^ bacteria/mL) with and without ASA sugar pretreatment. (G) Number of infective Dengue units per midgut (PFU) in mosquitoes pre-treated with ASA for 2 days (Dengue New Guinea C strain). (A) to (F) Error bars represent mean ± SEM. (G) Red bars represent the median of each group. (A) to (F) Unpaired t-test, NS (P>0.05), *P≤0.05, **P≤0.01; Conditions were compared with its correspondent SF control. (A) to (E) Pools of 10 midguts were used for each biological replicate, at least 3 biological replicates were used per condition. (F) Mortality rate across 6 independent survival curves. (G) Number of PFU per mosquito midgut, each dot represents one individual mosquito (control = 69 and ASA = 74).

To confirm these observations in adult mosquitoes, we pretreated female mosquitoes with 5 mM ASA in a sucrose solution for 3 days and fed or not with blood in order to allow growth of the indigenous microbiota. The same pattern observed for cells was observed in mosquitoes. Defensin A, cecropin G and cecropin D, which were down-regulated upon ASA treatment in the microarray analysis, had their expression increased in response to the microbiota growth, but treatment of the females with ASA prevented the up-regulation of this AMPs expression (Fig 3A–3C). Expression of serpin 27A and gambicin were independent of prostaglandin production, both in cells and mosquitoes, and treatment with ASA did not alter their expression in comparison to blood-fed non-ASA treated females as observed in the microarray (Fig 3D and 3E).

To evaluate whether impairment of prostaglandin production would also compromise the efficiency of the immune response in mosquitoes, *Aedes aegypti* females were allowed to feed on a sugar solution containing 5 mM ASA for 3 days prior to feeding on a saline solution (BBSA) containing the entomopathogenic bacteria *Serratia marcescens* (5x10^8^ bacteria/mL) [32]. The control cohort was not treated with ASA. Mosquito survival was then monitored daily for five days as an indicator of the capacity to cope with bacterial infection and hence immunocompetence (Fig 3F). Treatment of mosquitoes with ASA compromised anti-bacterial defense by increasing the mortality rate from 28% to 49%, in the case of *Serratia marcescens* (Fig 3F). Hence, here we show that prostaglandin production is also required for the generation of a fully functional immune response in mosquitoes.

To evaluate if inhibition of PG synthesis with ASA would increase viral loads also in mosquitoes, *Aedes aegypti* female mosquitoes were pretreated with 5mM of ASA and then, infected with Dengue virus (New Guinea C strain). Seven days post infection, the cohort that received ASA treatment prior to infection presented a 6.5 fold increase in viral particles in comparison to ASA non treated group (Fig 3G).

### The expression of the cytosolic Phospholipase A2 (PLA2c) is sensitive to different immune challenges

**Fig 4:**
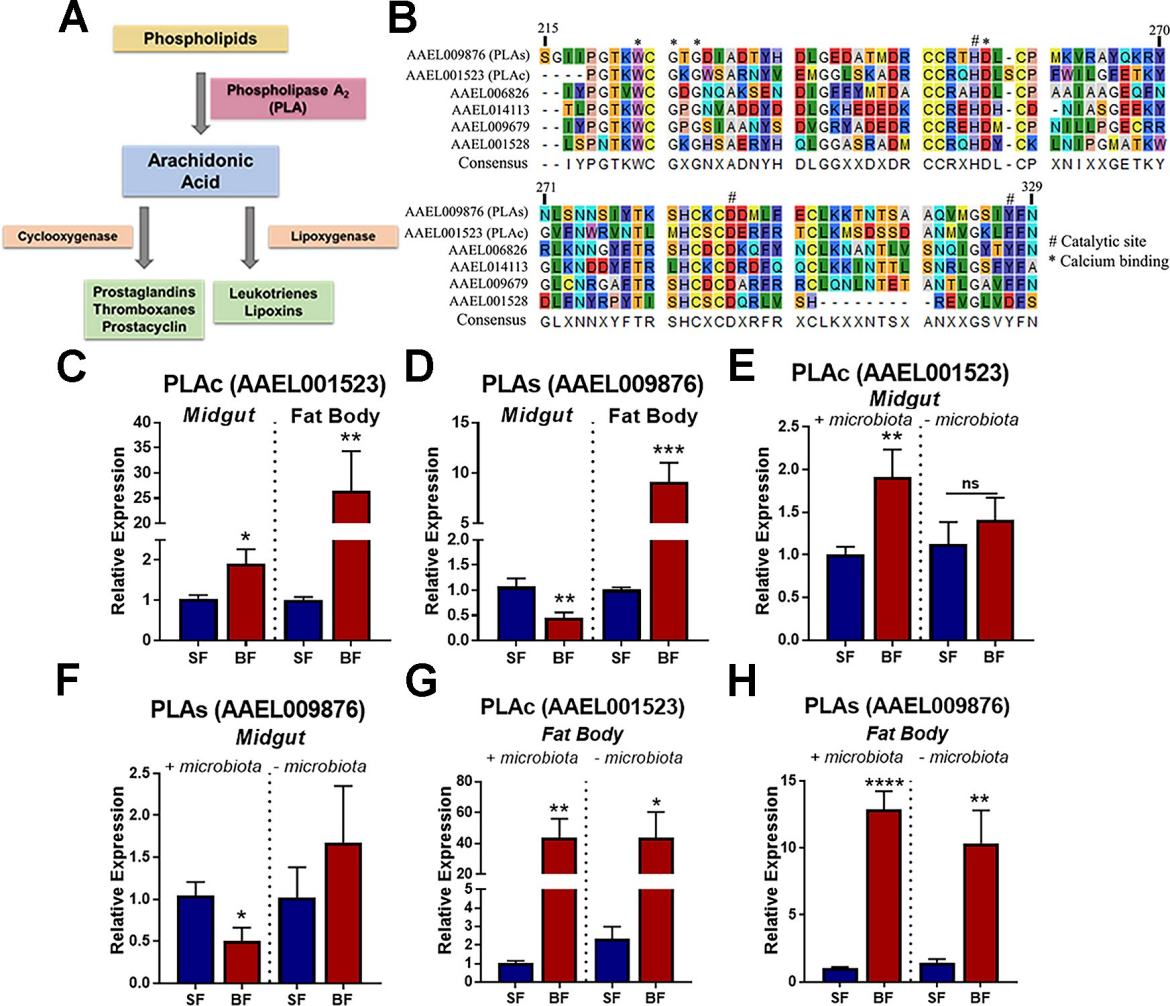
Cytosolic phospholipase A2 (PLA2c) is induced by the microbiota in the midgut after blood feeding. (A) Schematic representation of canonical PG production pathway. (B) Alignment of the amino acid sequences of six phospholipases of *Aedes aegypti*. * represents calcium binding domain; # represents catalytic domain. Gene expression of (C) PLA2c and (D) PLA2s in the midgut and fat body in sugar fed (SF) and blood fed mosquitoes (BF), 24 h post feeding. Gene expression of (E) PLA2c and (F) PLA2s in the midgut with or without the presence of the microbiota. Gene expression of (G) PLA2c and (H) PLA2s in the fat body with or without the microbiota presence. Error bars represent mean ± SEM. Unpaired t-test, NS (P>0.05), *P≤0.05, **P≤0.01, ***P≤0.001, ****P≤0.0001; Conditions were compared with its correspondent SF control. Each biological replicate was a pool of 10 midguts, and each experimental group had at least 3 biological replicates.

To further investigate the role of prostaglandins in mosquito immune responses, we targeted components of prostaglandins synthesis pathway and identified in the microarray a cytosolic phospholipase A2 (PLA2c; AAEL001523) that was downregulated by ASA treatment in the presence of a bacterial challenge (S1 Dataset). Phospholipase A2 (PLA2) mediates the first step in eicosanoid synthesis by converting membrane phospholipids into arachidonic acid that will be converted into prostaglandins, leukotrienes, lipoxins and prostacyclins (Fig 4A). We analyzed the amino acid sequence of six annotated PLA2, including the candidate one, selected from the microarray. All the sequences had conserved residues in the catalytic domain and calcium binding sites (Fig 4B). We selected the PLA2c (AAEL001523) and a secretory PLA2 (PLA2s) (AAEL009876) for further biological characterization. Those candidates were classified as cytosolic and secreted based on the presence of a signal peptide, using signal analysis. Twenty-four hours post feeding, PLA2c was upregulated following a blood meal in the midgut and in the fat body (Fig 4C), while PLA2s was downregulated in the midgut, but upregulated in the fat body (Fig 4D). The upregulation of PLA2s in the midgut following a blood meal was dependent on the presence of the microbiota, since antibiotics treatment abolished its induction (Fig 4E). The microbiota reduction abolished the downregulation of PLA2s on the midgut (Fig 4F). The expression of both PLA2c and PLA2s in the fat body was not affected by the status of the microbiota (Fig 4G and 4H). We also measured PLA2s and PLA2c expression in Aag-2 cell line challenged with different stimulus: heat killed *Enterobacter cloacae* (gram-negative); *Micrococcus luteus* (gram-positive) and zymosan. PLA2c and PLA2s were induced upon bacterial challenge after 6 h of incubation but not with zymosan (S6A Fig). At 24 h, no PLA2c regulation was observed upon any of the challenges, while PLA2s was induced by heat killed gram-negative bacteria and zymosan (S6B Fig).

### The cytosolic Phospholipase A2 modulates the expression of prostaglandin and protects against Dengue infection

**Fig 5:**
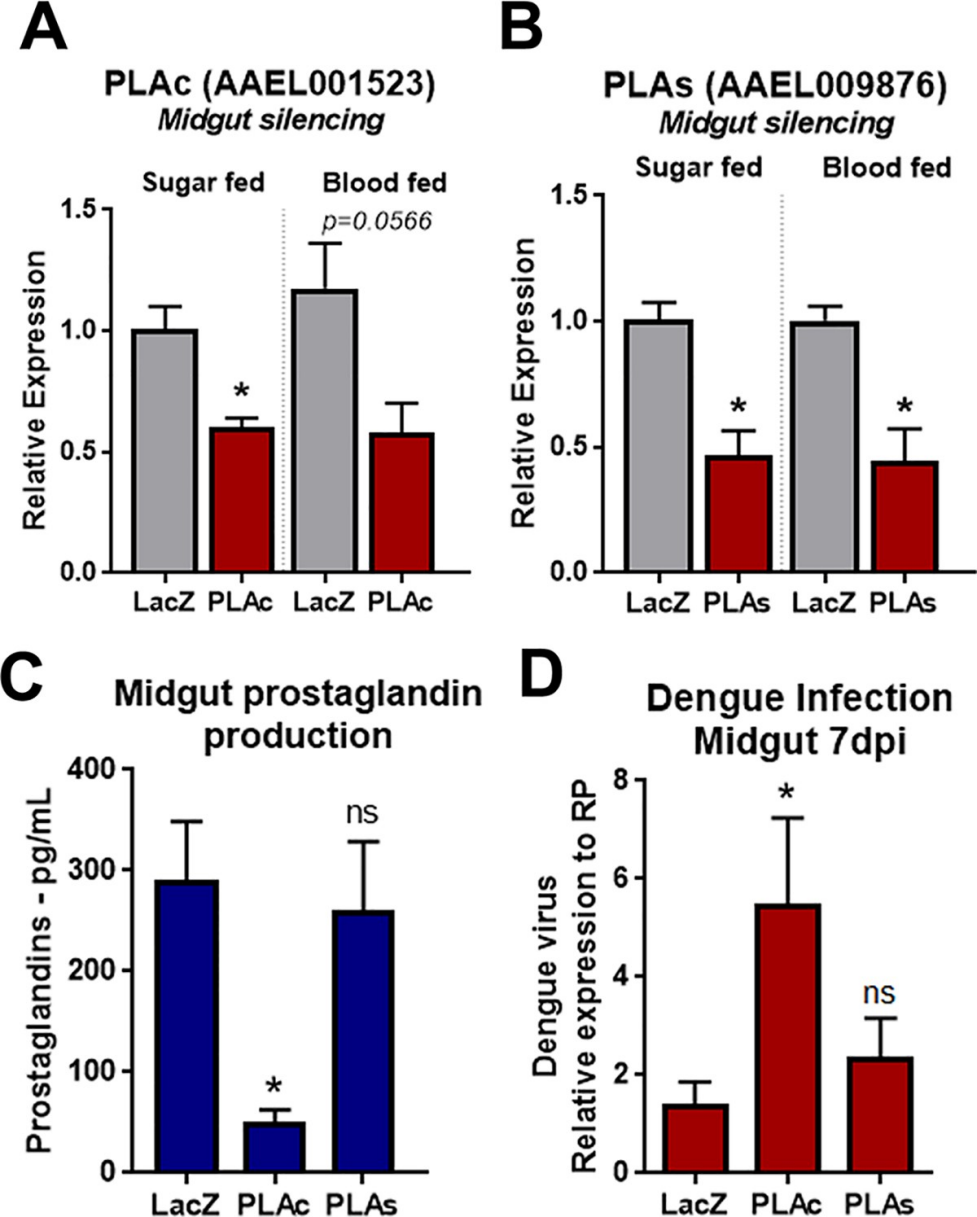
PG production in the midgut is dependent on PLA2c expression and its impairment increases Dengue viral loads. Knock-down efficiency in the mosquito midgut injected with (A) dsPLAc dsRNA and (B) dsPLAs dsRNA, in sugar fed (SF) and blood fed (BF) females. (C) PG levels in the midgut after RNAi silencing of PLAc and PLAs. Mosquitoes injected with dsRNA for LacZ were used as control. (D) Dengue virus RNA levels in the midgut relative to mosquito RP-49 expression. Error bars represent mean ± SEM. Unpaired t-test, NS (P>0.05) and *P≤0.05. Each biological replicate was a pool of 10 midguts, and each experimental group had at least 3 biological replicates.

Next, to evaluate the functional role of phospholipases in midgut prostaglandin production, we knocked down PLA2c and PLA2s expression using RNAi. Three days after dsRNA injection, PLA2c and PLA2s expression had a 50% reduction in expression, both in sugar and blood fed conditions (Fig 5A and 5B). The silencing of PLA2s significantly decreased in 70% the production of PG in the midgut, whereas the silencing of PLA2c did not affect PGs production (Fig 5C). We evaluated whether phospholipases and PG production played a role on Dengue infection in the midgut. The knockdown of PLA2c increased the Dengue viral loads (DV2) in the midgut over 5-fold compared to LacZ, whereas PLA2s silencing had no effect on viral loads (Fig 5D).

## Discussion

We show that, upon incubation with gram negative bacteria *E*. *cloacae*, Aag2 cells increase PG production in at least 10-fold, being this PG increase prevented by pre-incubation of the cells with ASA, a cyclooxygenase inhibitor (Fig 1A and 1B). Similarly, when mosquitoes are fed on a sugar solution containing antibiotics, PG levels were also reduced in more than 60-fold (Fig 1C), supporting the hypothesis that in mosquitoes, the microbiota induces the production of PG by the midgut. Previously, PGs were described as key molecules released by the midgut of *Anopheles gambia*e in response to the direct contact of the microbiota with the midgut epithelia. Not only PGs have a chemotactic effect on hemocytes but are also essential for immune memory establishment after *Plasmodium berghei* infection [11].

In vertebrates, the main cellular sites of prostaglandin production are the perinuclear membrane, lipid bodies, phagosomes and endoplasmic reticulum [33]. We demonstrated that, in Aag-2 cells, prostaglandins are mainly synthetized in the nuclear and perinuclear region upon bacterial stimuli (Fig 1D).

The microarray analysis revealed that the upregulation of immune genes induced by Ec challenge is no longer observed if the cells were pretreated with ASA. The expression of effector molecules such as cecropins, defensin, serpins; transcription factors, like REL 2; and regulators of the main immune pathways, such as SOCs and Cactus (IκB homolog), is reduced when prostaglandin synthesis is blocked (Fig 2A). This suggests a global immunosuppression of gene expression of several key immune genes upon ASA treatment even in the presence of bacterial challenge. In the mosquito *Anopheles albimanus*, antimicrobial peptides expression in the midgut is reduced upon eicosanoid inhibition and reverted when PGE_2_ is added to the media [14]. Although affecting a wide range of classes of immune genes, from PAMP receptors and intracellular signaling to effector genes, the effect of ASA treatment seems specific, since several immune genes, such as gambicin and serpin27A are not modulated when prostaglandin synthesis is inhibited (Figs 3A–3E and S3). ASA treatment does not prevent the induction of immune genes expression. Instead, the lack of PGs just reduces the amplitude of the response. In vertebrates, PGE_2_ has different effects depending on the cell type, it supports acute local inflammation and phagocyte attraction, but also has a suppressive role to control type-1 immune responses and host self-preservation [34]. In the insect *Spodoptera exigua* (beet armyworm), PGE_2_ synthesis inhibition suppresses phenoloxidase activity and reduces AMPs expression levels [10]. We confirmed that the inhibition of PG synthesis suppresses several immune genes, including AMPs, leading to a more permissive environment for bacterial and viral replication in mosquitoes. Thus, it seems that in insects, depletion of PGs promotes immune suppression, reducing the effectiveness of the immune response against bacteria and virus.

The microarray findings using Aag2 cells were confirmed by feeding female mosquitoes with ASA in the sugar followed by a blood meal. The lack of prostaglandins prevents the upregulation of defensin, cecropins D and G, while Serpin 27A and gambicin’s expression were not sensitive to ASA treatment. Treating Aag2 cells with another inhibitor of PG synthesis, Ibuprofen, a compound that inhibits cyclooxygenase through a different mechanism from ASA, also resulted in antimicrobial peptides expression down-regulation and increase in pathogen replication, for both Ec and Dengue. Thus, it is unlikely that effects observed with ASA treatment constitute off-target effects of this compound (S4 Fig).

Global immune down-regulation due to PG synthesis inhibition creates a permissive environment for pathogen replication and spread, since those pathways have previously been implicated in the clearance of *Plasmodium sp* and Dengue virus [24, 25, 35, 36]. Inhibition of PG synthesis turns Aag2 cells more permissive to a flavivirus, Dengue-2, to an alphavirus, Sindbis, and to the bacteria Enterobacter cloacae (Fig 2B–2D and S5 Fig). The cells became unable to control infections with multiple pathogens, indicating that PGs affect the proper activation of a general mechanism of fighting infections. Mosquitoes pretreated with ASA in the sugar were also more permissive to Dengue infection in the midgut reproducing the findings obtained with Aag2 cells (Figs 2C and 3G, S5B and S5C Fig). Overall, impairment of PG synthesis leads to a decreased effectiveness in viral replication control, caused by the low expression of immune related genes.

The loss of the ability to control pathogen replication can lead to a decrease in host survival, especially upon bacterial infections where its overgrowth compromises the life of the insect. According to this, mosquitoes pretreated with ASA were more sensitive to Serratia infection and had a mortality rate higher than mosquitoes not treated and infected with the bacteria (Fig 3F). This suggests that the immunosuppression caused by the lack PG production leads to pathogen susceptibility and results in survival reduction.

Although PGs can be detected in mosquitoes [37], insects lack a canonical pathway of PG synthesis, lacking a direct ortholog of cyclooxygenase. A recent study described that heme peroxidases, HPX7 and HPX8, are involved and necessary for PG synthesis in the mosquito *Anopheles gambiae* [11]. Microbiota proliferation induces the expression of HPX7 and HPX8 that results in PG production in the midgut. Here we decided to investigate the upstream components of eicosanoid production, by characterizing the phospholipase A2 that converts phospholipids into arachidonic acid, the first reaction of the pathway. Our results describe a phospholipase directly related to PG synthesis, unraveling one more step of this pathway in mosquitoes. Although the two PLA2 tested have a similar amino acid sequence, especially in catalytic and Ca+2 binding sites, they appear to have distinct biological roles in mosquito physiology. The two phospholipases, PLA2c and PLA2s, were upregulated in the fat body in response to blood feeding (Fig 4C, 4D, 4G and 4H). That could indicate lipid mobilization and secretion in the hemolymph for egg development after the nutritional input of the blood ingestion [38]. Only PLA2c was upregulated in the midgut in response to the blood meal and this increase was microbiota dependent, indicating that bacterial elicitors might be triggering the expression in the midgut (Fig 4C–4F). This could argue that this PLA2c is playing an immune role in the midgut, while the other PLA2s seems to have a stronger regulation in the fat body independently of the microbiota presence. Using gene knockdown through RNAi both PLA2c and PLA2s were successfully silenced in the midgut in both sugar and blood fed mosquitoes (Fig 5A and 5B). Knockdown of PLA2c resulted in the decrease of PG production in the midgut and higher amounts of Dengue RNA (Fig 5C and 5D). Previously, it has been shown that PG levels increase during Dengue infection in the midgut of *Ae*. *aegypti* mosquitoes [37]. PG increase during Dengue infection could indicate an attempt of the mosquito immune response to control viral replication. When PG synthesis is blocked, by either ASA or Ibuprofen treatments or PLA2c knockdown, the mosquito becomes more susceptible to viral replication.

Altogether, our results indicate that prostaglandins are an important component of the immune response in mosquitoes, not being responsible for the activation of the immune response but playing a role in the amplitude of this response. We shed some light on how PGs are responsible for this “fine tuning” of the immune response by providing a genome wide analysis of the effects of PG on *Aedes* gene expression, revealing that PGs may modulate the expression of several genes from the main mosquito immune pathways, Toll, IMD and Jak/STAT, and probably, as a consequence of this modulation, alter the expression of effector genes. We described a PLA2c that is involved in PG synthesis in the midgut and showed that, when it is silenced, Dengue replication increases. Here we showed using pharmacological and genetic approaches the role of PGs in immune modulation and viral susceptibility in the mosquito *Ae*. *aegypti*.

## Supporting information

S1 Fig Bacterial 16S expression in the midgut of Ae. aegypti upon antibiotic treatment. Quantitative PCR of bacterial 16S mRNA in midguts from sugar (SF) and blood fed (BF) mosquitoes kept on regular sugar or treated for four days with antibiotics (+Ab). Expression was evaluated 24 hours post blood feeding. 16S expression was calculated relative to the mosquito ribosomal protein 49 (RP49) used as an endogenous control.
(TIF)

S2 Fig Venn diagram of the microarray analysis of Aag2 cells stimulated with *E*. *cloacae (*Gram Negative, Ec) in the presence of ASA. 361 genes were up-regulated in response to Ec incubation, while 822 were down-regulated. In the presence of Ec and ASA 1008 genes were up-regulated and 1267 were down-regulated. 114 genes were up-regulated in both conditions while 399 were down-regulated. For detailed information on the genes see S1 Dataset.
(TIF)

S3 Fig ASA treatment impairs AMPs expression in response to bacterial challenge. Gene expression of AMPs identified in the microarray analysis in Aag2 cells challenged with heat-killed Ec in the presence of ASA. (A) Defensin A, (B) Cecropin G, (C) Cecropin D, (D) Attacin and (E) Gambicin. Error bars represent mean ± SEM. ANOVA Dunn’s multiple comparison test, NS (P>0.05), *P≤0.05, **P≤0.01, ****P≤0.0001. Each biological replicate was an individual well from a culture plate, and each experimental group had at least 3 biological replicates. AMPs expression was calculated relative to the expression of the RP-49 gene.
(TIF)

S4 Fig Inhibition of PG production with ibuprofen leads to a decrease in several immune-related genes after bacterial infection in Aag2 cells. Gene expression of AMPs in response to heat-killed Ec challenge in the presence of ibuprofen. (A) Defensin A and (B) Gambicin. Error bars represent mean ± SEM. ANOVA Dunn’s multiple comparison test, NS (P>0.05), ***P≤0.001. Each biological replicate was an individual well from a culture plate, and each experimental group had at least 3 biological replicates. AMPs expression was calculated relative to the expression of the RP-49 gene.
(TIF)

S5 Fig In the absence of PG, Aag2 cells capacity of controlling E. cloacae or Dengue virus proliferation is compromised. Aag2 cells were challenged with live Ec and Dengue virus in the presence of prostaglandin synthesis inhibitors, ibuprofen and ASA. (A) Number of CFU in the supernatant of challenged cells incubated with ibuprofen. (B) Viral RNA present in the supernatant of cells infected with *New Guinea C* Dengue 2 strain in the presence of ASA. (C) Viral RNA present in the supernatant of cells infected with *Halstead* Dengue 2 strain in the presence of ibuprofen. Viral RNA amounts in present in the supernatant of the cell culture were normalized by the number of cells present in the well, which were determined using trypan blue stain. Error bars represent mean ± SEM. Unpaired t-test, *P≤0.05, **P≤0.01. Each biological replicate was an individual well from a culture plate, and each experimental group had at least 3 biological replicates.
(TIF)

S6 Fig Both cytosolic and secretory PLA are transcriptionally regulated by bacterial and fungal products. Aag2 cells challenged with heat-killed Gram positive (Ml) and negative (Ec) bacteria and zymosan (Zy) a glucan present in fungus surface. Cells were challenged for 6 and 24 hours. (A) Gene expression of PLAc 6 and 24 hours post stimulus. (B) Gene expression of PLAs 6 and 24 hours post stimulus. Error bars represent mean ± SEM. Dunn’s multiple comparison test, NS (P>0.05), *P≤0.05, **P≤0.01, ***P≤0.001, ****P≤0.0001. Each biological replicate was an individual well from a culture plate, and each experimental group had at least 3 biological replicates. PLAc and PLAs expression were normalized using the expression of endogenous RP-49 gene.
(TIF)

S1 Table List of primers used in this study. (TIF)

S1 Dataset Gene expression changes of Aag2 cells modulated by bacterial infection in the presence or absence of ASA. (XLSX)

S2 Dataset Immune genes modulated by ASA treatment. (XLSX)

## References

[1] Kumar A, Srivastava P, Sirisena P, Dubey SK, Kumar R, Shrinet J, et al Mosquito Innate Immunity. Insects. 2018;9(3). Epub 2018/08/12. 10.3390/insects9030095 30096752 PMC6165528

[2] Lemaitre B, Hoffmann J. The host defense of Drosophila melanogaster. Annu Rev Immunol. 2007;25:697–743. Epub 2007/01/05. 10.1146/annurev.immunol.25.022106.141615 . 17201680

[3] Buchon N, Silverman N, Cherry S. Immunity in Drosophila melanogaster—from microbial recognition to whole-organism physiology. Nat Rev Immunol. 2014;14(12):796–810. Epub 2014/11/26. 10.1038/nri3763 25421701 PMC6190593

[4] Gupta L, Molina-Cruz A, Kumar S, Rodrigues J, Dixit R, Zamora RE, et al The STAT pathway mediates late-phase immunity against Plasmodium in the mosquito Anopheles gambiae. Cell Host Microbe. 2009;5(5):498–507. Epub 2009/05/21. 10.1016/j.chom.2009.04.003 19454353 PMC2701194

[5] Destephano DB, Brady UE, Woodall LB. Partial characterization of prostaglandin synthetase in the reproductive tract of the male house cricket, Acheta domesticus. Prostaglandins. 1976;11(2):261–73. Epub 1976/02/01. . 4855

[6] Stanley-Samuelson D, Jurenka RA, Blomquist GJ, Loher W. De novo biosynthesis of prostaglandins by the Australian field cricket, Teleogryllus commodus. Comp Biochem Physiol C. 1986;85(2):303–7. Epub 1986/01/01. 10.1016/0742-8413(86)90198-2 . 2879689

[7] Stanley-Samuelson DW, Jensen E, Nickerson KW, Tiebel K, Ogg CL, Howard RW. Insect immune response to bacterial infection is mediated by eicosanoids. Proc Natl Acad Sci U S A. 1991;88(3):1064–8. Epub 1991/02/01. 10.1073/pnas.88.3.1064 1899480 PMC50955

[8] Miller JS, Nguyen T, Stanley-Samuelson DW. Eicosanoids mediate insect nodulation responses to bacterial infections. Proc Natl Acad Sci U S A. 1994;91(26):12418–22. Epub 1994/12/20. 10.1073/pnas.91.26.12418 7809052 PMC45449

[9] Azambuja P, Ratcliffe NA, Garcia ES. Towards an understanding of the interactions of Trypanosoma cruzi and Trypanosoma rangeli within the reduviid insect host Rhodnius prolixus. An Acad Bras Cienc. 2005;77(3):397–404. Epub 2005/08/30. 10.1590/s0001-37652005000300004 . 16127548

[10] Ahmed S, Stanley D, Kim Y. An Insect Prostaglandin E2 Synthase Acts in Immunity and Reproduction. Front Physiol. 2018;9:1231 Epub 2018/09/21. 10.3389/fphys.2018.01231 30233407 PMC6131586

[11] Barletta ABF, Trisnadi N, Ramirez JL, Barillas-Mury C. Mosquito Midgut Prostaglandin Release Establishes Systemic Immune Priming. iScience. 2019;19:54–62. Epub 2019/07/28. 10.1016/j.isci.2019.07.012 31351392 PMC6661395

[12] Petzel DH, Stanleysamuelson DW. Inhibition of Eicosanoid Biosynthesis Modulates Basal Fluid Secretion in the Malpighian Tubules of the Yellow-Fever Mosquito (Aedes-Aegypti). J Insect Physiol. 1992;38(1):1–8. 10.1016/0022-1910(92)90016-7 WOS:A1992HG54700001.

[13] Qian Y, Essenberg RC, Dillwith JW, Bowman AS, Sauer JR. A specific prostaglandin E2 receptor and its role in modulating salivary secretion in the female tick, Amblyomma americanum (L.). Insect Biochem Mol Biol. 1997;27(5):387–95. Epub 1997/05/01. 10.1016/s0965-1748(97)00010-6 . 9219365

[14] Garcia Gil de Munoz FL, Martinez-Barnetche J, Lanz-Mendoza H, Rodriguez MH, Hernandez-Hernandez FC. Prostaglandin E2 modulates the expression of antimicrobial peptides in the fat body and midgut of Anopheles albimanus. Arch Insect Biochem Physiol. 2008;68(1):14–25. Epub 2008/04/17. 10.1002/arch.20232 . 18412259

[15] Kwon H, Yang Y, Kumar S, Lee DW, Bajracharya P, Calkins TL, et al Characterization of the first insect prostaglandin (PGE2) receptor: MansePGE2R is expressed in oenocytoids and lipoteichoic acid (LTA) increases transcript expression. Insect Biochem Mol Biol. 2020;117:103290 Epub 2019/12/04. 10.1016/j.ibmb.2019.103290 . 31790798

[16] Tithof PK, Roberts MP, Guan W, Elgayyar M, Godkin JD. Distinct phospholipase A2 enzymes regulate prostaglandin E2 and F2alpha production by bovine endometrial epithelial cells. Reprod Biol Endocrinol. 2007;5:16 Epub 2007/04/27. 10.1186/1477-7827-5-16 17459165 PMC1868772

[17] Stanley D. Prostaglandins and other eicosanoids in insects: biological significance. Annu Rev Entomol. 2006;51:25–44. Epub 2005/12/08. 10.1146/annurev.ento.51.110104.151021 . 16332202

[18] Varvas K, Kurg R, Hansen K, Jarving R, Jarving I, Valmsen K, et al Direct evidence of the cyclooxygenase pathway of prostaglandin synthesis in arthropods: genetic and biochemical characterization of two crustacean cyclooxygenases. Insect Biochem Mol Biol. 2009;39(12):851–60. Epub 2009/10/27. 10.1016/j.ibmb.2009.10.002 . 19854273

[19] Tootle TL, Spradling AC. Drosophila Pxt: a cyclooxygenase-like facilitator of follicle maturation. Development. 2008;135(5):839–47. Epub 2008/01/25. 10.1242/dev.017590 18216169 PMC2818214

[20] Gao Y, Hernandez VP, Fallon AM. Immunity proteins from mosquito cell lines include three defensin A isoforms from Aedes aegypti and a defensin D from Aedes albopictus. Insect Mol Biol. 1999;8(3):311–8. Epub 1999/09/01. 10.1046/j.1365-2583.1999.83119.x . 10469248

[21] Sung HH, Chang HJ, Her CH, Chang JC, Song YL. Phenoloxidase activity of hemocytes derived from Penaeus monodon and Macrobrachium rosenbergii. J Invertebr Pathol. 1998;71(1):26–33. Epub 1998/02/03. 10.1006/jipa.1997.4703 . 9446734

[22] Mukherjee S, Hanley KA. RNA interference modulates replication of dengue virus in Drosophila melanogaster cells. BMC Microbiol. 2010;10:127 Epub 2010/04/28. 10.1186/1471-2180-10-127 20420715 PMC2874549

[23] Oliveira JH, Goncalves RL, Lara FA, Dias FA, Gandara AC, Menna-Barreto RF, et al Blood meal-derived heme decreases ROS levels in the midgut of Aedes aegypti and allows proliferation of intestinal microbiota. PLoS Pathog. 2011;7(3):e1001320 Epub 2011/03/30. 10.1371/journal.ppat.1001320 21445237 PMC3060171

[24] Xi Z, Ramirez JL, Dimopoulos G. The Aedes aegypti toll pathway controls dengue virus infection. PLoS Pathog. 2008;4(7):e1000098 Epub 2008/07/08. 10.1371/journal.ppat.1000098 18604274 PMC2435278

[25] Souza-Neto JA, Sim S, Dimopoulos G. An evolutionary conserved function of the JAK-STAT pathway in anti-dengue defense. Proc Natl Acad Sci U S A. 2009;106(42):17841–6. Epub 2009/10/07. 10.1073/pnas.0905006106 19805194 PMC2764916

[26] Yang IV, Chen E, Hasseman JP, Liang W, Frank BC, Wang S, et al Within the fold: assessing differential expression measures and reproducibility in microarray assays. Genome Biol. 2002;3(11):research0062 Epub 2002/11/14. 10.1186/gb-2002-3-11-research0062 12429061 PMC133446

[27] Gentile C, Lima JB, Peixoto AA. Isolation of a fragment homologous to the rp49 constitutive gene of Drosophila in the Neotropical malaria vector Anopheles aquasalis (Diptera: Culicidae). Mem Inst Oswaldo Cruz. 2005;100(6):545–7. Epub 2005/11/23. 10.1590/s0074-02762005000600008 . 16302065

[28] Bandeira-Melo C, Paiva LA, Amorim NRT, Weller PF, Bozza PT. EicosaCell: An Imaging-Based Assay to Identify Spatiotemporal Eicosanoid Synthesis. Methods Mol Biol. 2017;1554:127–41. Epub 2017/02/12. 10.1007/978-1-4939-6759-9_6 28185186 PMC5774667

[29] Gusmao DS, Santos AV, Marini DC, Bacci M Jr., Berbert-Molina MA, Lemos FJ. Culture-dependent and culture-independent characterization of microorganisms associated with Aedes aegypti (Diptera: Culicidae) (L.) and dynamics of bacterial colonization in the midgut. Acta Trop. 2010;115(3):275–81. Epub 2010/05/04. 10.1016/j.actatropica.2010.04.011 . 20434424

[30] Gaio Ade O, Gusmao DS, Santos AV, Berbert-Molina MA, Pimenta PF, Lemos FJ. Contribution of midgut bacteria to blood digestion and egg production in aedes aegypti (diptera: culicidae) (L.). Parasit Vectors. 2011;4:105 Epub 2011/06/16. 10.1186/1756-3305-4-105 21672186 PMC3125380

[31] Ramirez JL, Souza-Neto J, Torres Cosme R, Rovira J, Ortiz A, Pascale JM, et al Reciprocal tripartite interactions between the Aedes aegypti midgut microbiota, innate immune system and dengue virus influences vector competence. PLoS Negl Trop Dis. 2012;6(3):e1561 Epub 2012/03/14. 10.1371/journal.pntd.0001561 22413032 PMC3295821

[32] Castro DP, Seabra SH, Garcia ES, de Souza W, Azambuja P. Trypanosoma cruzi: ultrastructural studies of adhesion, lysis and biofilm formation by Serratia marcescens. Exp Parasitol. 2007;117(2):201–7. Epub 2007/06/16. 10.1016/j.exppara.2007.04.014 . 17570364

[33] Bozza PT, Bakker-Abreu I, Navarro-Xavier RA, Bandeira-Melo C. Lipid body function in eicosanoid synthesis: an update. Prostaglandins Leukot Essent Fatty Acids. 2011;85(5):205–13. Epub 2011/05/14. 10.1016/j.plefa.2011.04.020 . 21565480

[34] Kalinski P. Regulation of immune responses by prostaglandin E2. J Immunol. 2012;188(1):21–8. Epub 2011/12/22. 10.4049/jimmunol.1101029 22187483 PMC3249979

[35] Blandin S, Levashina EA. Mosquito immune responses against malaria parasites. Curr Opin Immunol. 2004;16(1):16–20. Epub 2004/01/22. 10.1016/j.coi.2003.11.010 . 14734105

[36] Clayton AM, Dong Y, Dimopoulos G. The Anopheles innate immune system in the defense against malaria infection. J Innate Immun. 2014;6(2):169–81. Epub 2013/08/31. 10.1159/000353602 23988482 PMC3939431

[37] Chotiwan N, Andre BG, Sanchez-Vargas I, Islam MN, Grabowski JM, Hopf-Jannasch A, et al Dynamic remodeling of lipids coincides with dengue virus replication in the midgut of Aedes aegypti mosquitoes. PLoS Pathog. 2018;14(2):e1006853 Epub 2018/02/16. 10.1371/journal.ppat.1006853 29447265 PMC5814098

[38] Arrese EL, Soulages JL. Insect fat body: energy, metabolism, and regulation. Annu Rev Entomol. 2010;55:207–25. Epub 2009/09/04. 10.1146/annurev-ento-112408-085356 19725772 PMC3075550

