# Supplementary figures and images for: Prostaglandins regulate humoral immune responses in Aedes aegypti
Source: PLoS Negl Trop Dis. 2020 Oct 23;14(10):e0008706. doi: 10.1371/journal.pntd.0008706 (PMC7584201; doi:10.1371/journal.pntd.0008706)

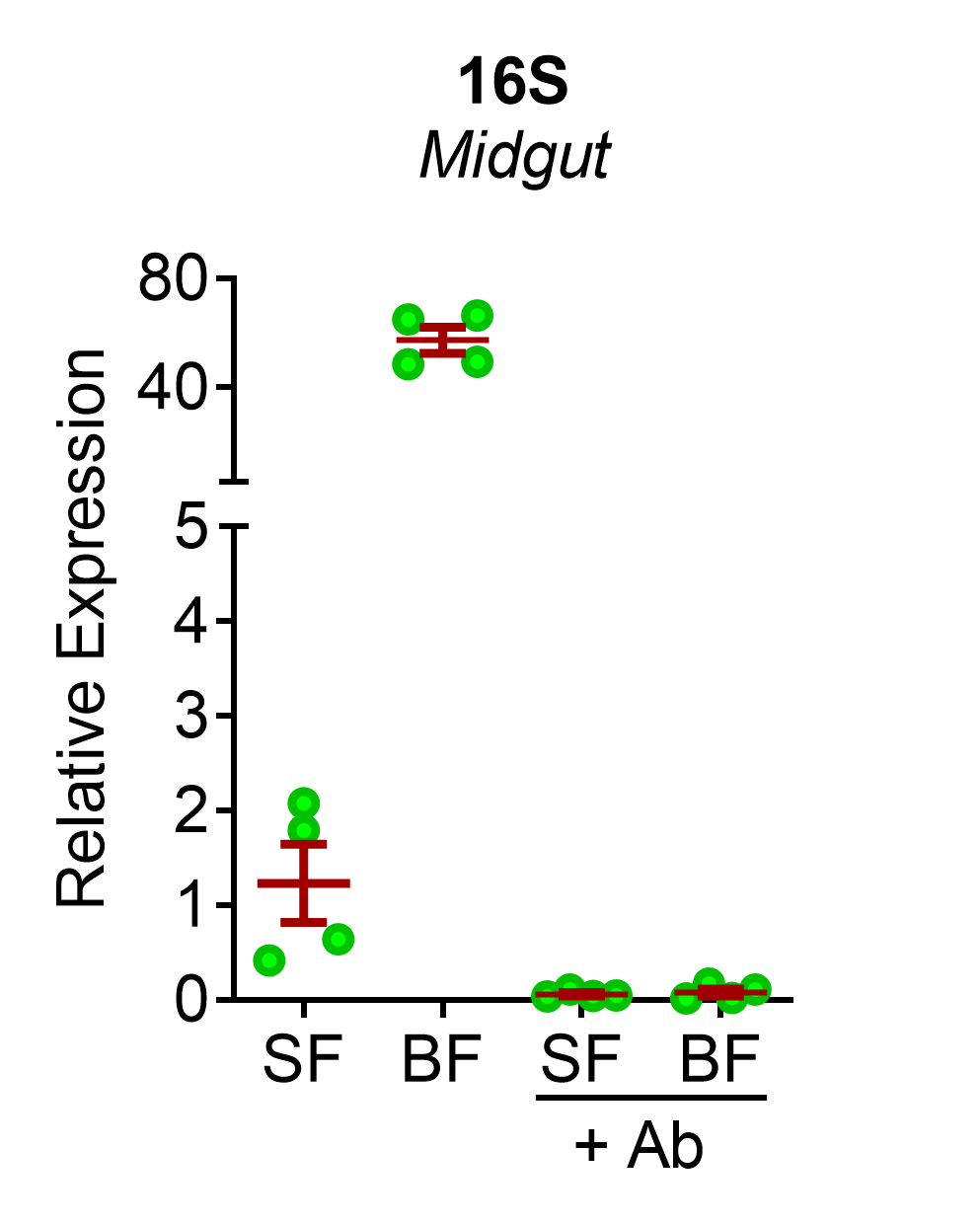

Supplement: S1 Fig — Quantitative PCR of bacterial 16S mRNA in midguts from sugar (SF) and blood fed (BF) mosquitoes kept on regular sugar or treated for four days with antibiotics (+Ab). Expression was evaluated 24 hours post blood feeding. 16S expression was calculated relative to the mosquito ribosomal protein 49 (RP49) used as an endogenous control. (TIF) [file pntd.0008706.s001.tif]

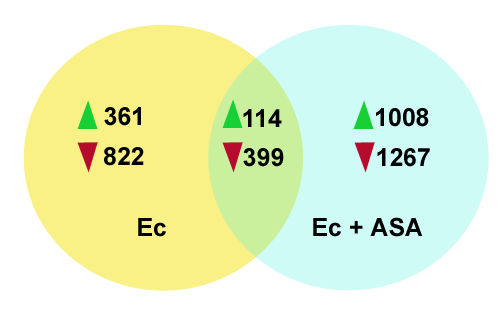

Supplement: S2 Fig — 361 genes were up-regulated in response to Ec incubation, while 822 were down-regulated. In the presence of Ec and ASA 1008 genes were up-regulated and 1267 were down-regulated. 114 genes were up-regulated in both conditions while 399 were down-regulated. For detailed information on the genes see S1 Dataset. (TIF) [file pntd.0008706.s002.tif]

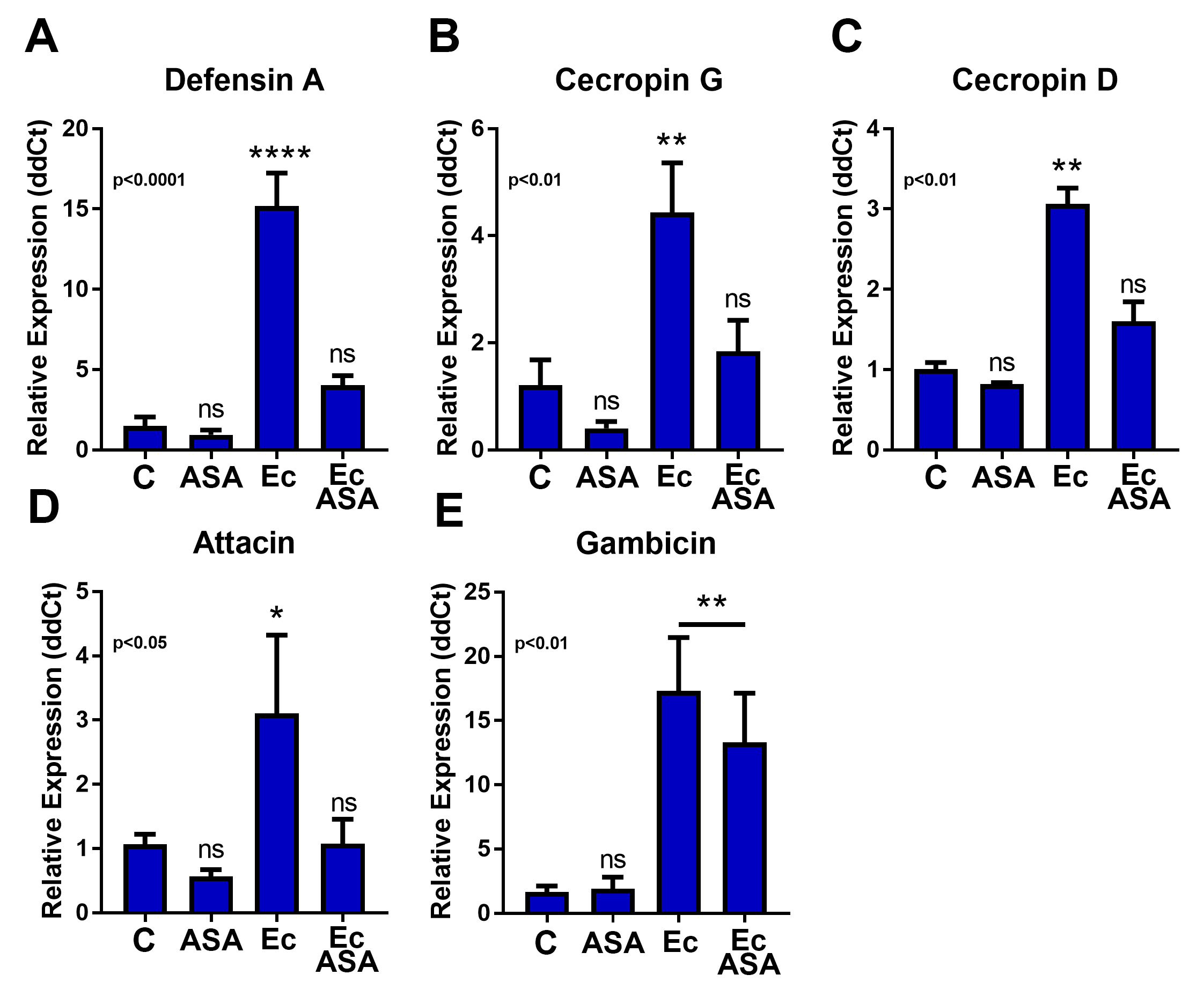

Supplement: S3 Fig — Gene expression of AMPs identified in the microarray analysis in Aag2 cells challenged with heat-killed Ec in the presence of ASA. (A) Defensin A, (B) Cecropin G, (C) Cecropin D, (D) Attacin and (E) Gambicin. Error bars represent mean ± SEM. ANOVA Dunn’s multiple comparison test, NS (P>0.05), *P≤0.05, **P≤0.01, ****P≤0.0001. Each biological replicate was an individual well from a culture plate, and each experimental group had at least 3 biological replicates. AMPs expression was calculated relative to the expression of the RP-49 gene. (TIF) [file pntd.0008706.s003.tif]

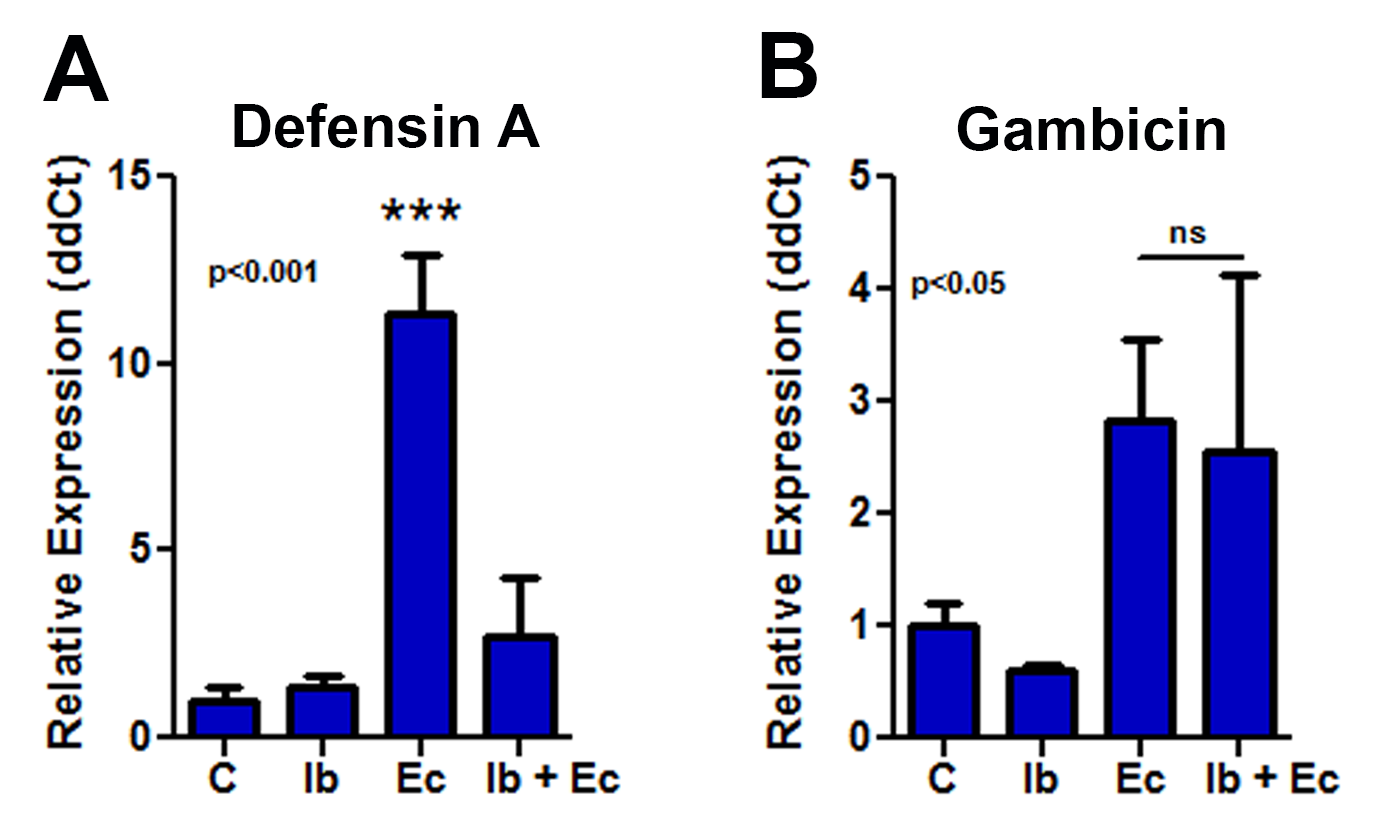

Supplement: S4 Fig — Gene expression of AMPs in response to heat-killed Ec challenge in the presence of ibuprofen. (A) Defensin A and (B) Gambicin. Error bars represent mean ± SEM. ANOVA Dunn’s multiple comparison test, NS (P>0.05), ***P≤0.001. Each biological replicate was an individual well from a culture plate, and each experimental group had at least 3 biological replicates. AMPs expression was calculated relative to the expression of the RP-49 gene. (TIF) [file pntd.0008706.s004.tif]

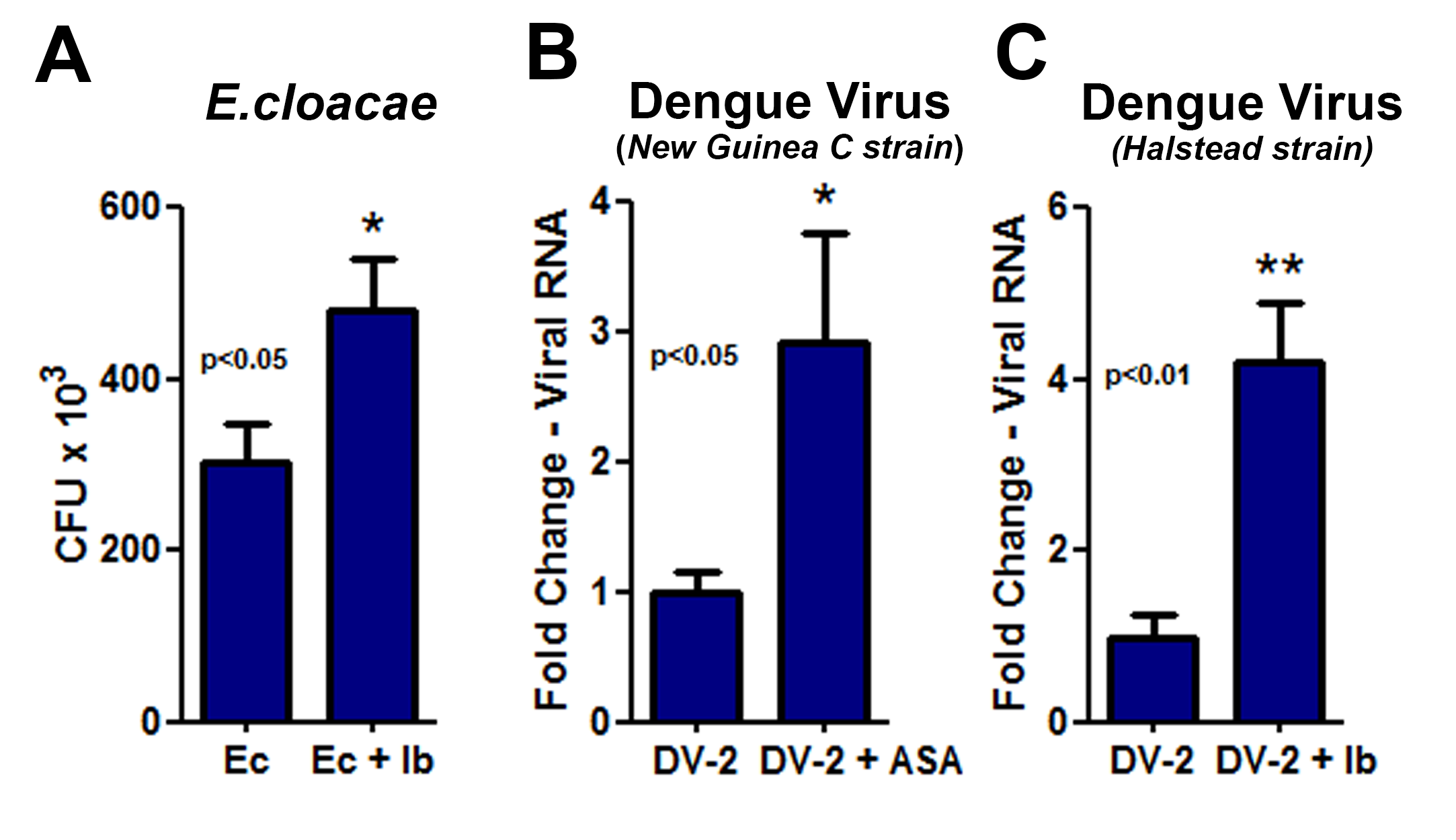

Supplement: S5 Fig — Aag2 cells were challenged with live Ec and Dengue virus in the presence of prostaglandin synthesis inhibitors, ibuprofen and ASA. (A) Number of CFU in the supernatant of challenged cells incubated with ibuprofen. (B) Viral RNA present in the supernatant of cells infected with New Guinea C Dengue 2 strain in the presence of ASA. (C) Viral RNA present in the supernatant of cells infected with Halstead Dengue 2 strain in the presence of ibuprofen. Viral RNA amounts in present in the supernatant of the cell culture were normalized by the number of cells present in the well, which were determined using trypan blue stain. Error bars represent mean ± SEM. Unpaired t-test, *P≤0.05, **P≤0.01. Each biological replicate was an individual well from a culture plate, and each experimental group had at least 3 biological replicates. (TIF) [file pntd.0008706.s005.tif]

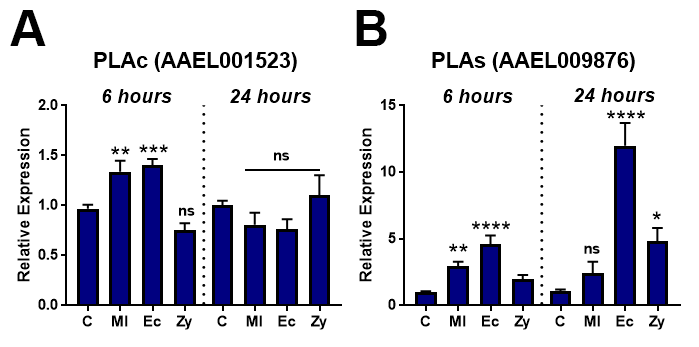

Supplement: S6 Fig — Aag2 cells challenged with heat-killed Gram positive (Ml) and negative (Ec) bacteria and zymosan (Zy) a glucan present in fungus surface. Cells were challenged for 6 and 24 hours. (A) Gene expression of PLAc 6 and 24 hours post stimulus. (B) Gene expression of PLAs 6 and 24 hours post stimulus. Error bars represent mean ± SEM. Dunn’s multiple comparison test, NS (P>0.05), *P≤0.05, **P≤0.01, ***P≤0.001, ****P≤0.0001. Each biological replicate was an individual well from a culture plate, and each experimental group had at least 3 biological replicates. PLAc and PLAs expression were normalized using the expression of endogenous RP-49 gene. (TIF) [file pntd.0008706.s006.tif]

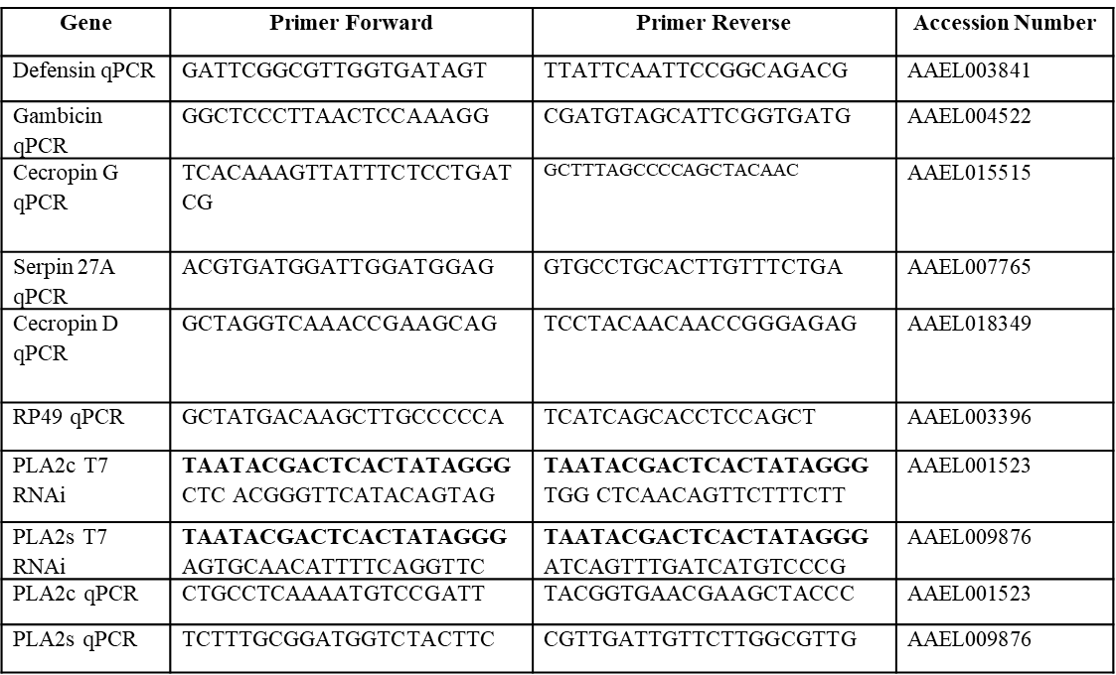

Supplement: S1 Table — (TIF) [file pntd.0008706.s007.tif]
